# Research progress of multi-enzyme complexes based on the design of scaffold protein

**DOI:** 10.1186/s40643-023-00695-8

**Published:** 2023-10-23

**Authors:** Xiangyi Wang, Yi Jiang, Hongling Liu, Haibo Yuan, Di Huang, Tengfei Wang

**Affiliations:** 1 State Key Laboratory of Biobased Material and Green Papermaking (LBMP), Qilu University of Technology (Shandong Academy of Sciences), Jinan, 250353 Shandong People’s Republic of China; 2 Key Laboratory of Shandong Microbial Engineering, School of Bioengineering, Qilu University of Technology (Shandong Academy of Sciences), Jinan, 250353 Shandong People’s Republic of China

**Keywords:** Enzyme cascades, Substrate channel, Scaffold protein, Fusion protein, Linker

## Abstract

Multi-enzyme complexes designed based on scaffold proteins are a current topic in molecular enzyme engineering. They have been gradually applied to increase the production of enzyme cascades, thereby achieving effective biosynthetic pathways. This paper reviews the recent progress in the design strategy and application of multi-enzyme complexes. First, the metabolic channels in the multi-enzyme complex have been introduced, and the construction strategies of the multi-enzyme complex emerging in recent years have been summarized. Then, the discovered enzyme cascades related to scaffold proteins are discussed, emphasizing on the influence of the linker on the fusion enzyme (fusion protein) and its possible mechanism. This review is expected to provide a more theoretical basis for the modification of multi-enzyme complexes and broaden their applications in synthetic biology.

## Key Points

- The strategies of compartmentalization and substrate channel for organisms to manipulate multi-enzyme reactions are reviewed.
- The discovered enzyme cascade reactions related to scaffold proteins are summarized.
- The influence of linkers on fusion proteins and their possible mechanisms are highlighted and prospected.

## Introduction

The catalytic efficiency of the cascade enzyme reaction in the multi-enzyme complex is influenced by various factors, such as the number of enzymes, spatial orientation, arrangement sequence, and linker (Argos 1990; Smith 2017; Sheng et al. 2022). An efficient multi-enzyme complex must optimally assemble enzymes from different sources and with different functions to form an ordered multi-enzyme complex. Many effective multi-enzyme assembly strategies have been established, including protein fusion technology, immobilization technology, and scaffold protein-mediated self-assembly technology.

At the genetic level, constructing a fusion enzyme is relatively simple and easy. However, there is no clear and complete standardized process for constructing of fusion protein in the current research, which solely relies on the experience of scientists. Moreover, the characteristics and functions of the constructed fusion protein multi-enzyme complex cannot be expected (Bülow L, 1991; Huang et al. 2021). Protein fusion technology enables the enzyme to form substrate channels in space, reducing substrate diffusion and by-product generation while improving the overall catalytic efficiency of the enzyme. Although fusion protein technology is a simple multi-enzyme assembly strategy, it also has drawbacks, including the inability of fusion enzyme expression, low protein expression, and protein misfolding to form inclusion bodies (Zhang 2011; Shi 2018; Arai 2021). We have a limited understanding of the impact of fusion protein structure, protein interaction, spatial orientation, linker flexibility, and protein distance on fusion proteins. Therefore, without causing the metabolic burden of host cells, the design of "quantitative, sequential and spatial" controllable multi-enzyme cascade reaction is a hot topic for international academic circles. Some researchers have been trying to design fusion proteins rationally through computer simulation, and changing the length and type of protein linker to solve the above problems (Robinson and Sauer 1998; Li et al. 2016). Immobilization of enzymes is an effective method for resolving their solubility and stability issues, allowing the enzymes to be reused and lowering the cost of the biological process (Schoffelen and van Hest 2012).

Scaffold protein is a new form that mimics the natural multi-enzyme complex cascade reaction. Some macromolecular protein complexes have a special protein–protein interaction. The scaffold proteins are defined as proteins that organize signal complexes by binding at least two signal enzymes together and facilitating their communication through proximity. Synthetic scaffolds are mainly used for soluble enzyme systems, with cellulosomes being the most typical example (Bayer 2008). Lamed et al. (Lamed 1983) purified a cellulose degradation-related protein complex from *C. thermocellum* for the first time in 1983, which was proved to be a cellulosome. Cellulosome is an extracellular multi-enzyme complex produced by some anaerobic bacteria in nature and formed by various cellulases that can efficiently degrade cellulose (Maki 2009). Notably, cellulosomes are divided into two parts: one part is a scaffold protein composed of multiple cohesins in varying orders and quantities, with the function of the assembly. Scaffold protein contains non-catalytic cellulose-binding modules (CBM), which can bind multi-enzyme complexes to cellulose (Bayer 2008; Zverlov 2008; Bule 2016). The other part is the catalytic module composed of a range of cellulases connected with dockerins with catalytic function. The two parts form a multi-enzyme complex by interacting with dockerins and cohesins. Dockerins and cohesins have specific interaction mechanisms between species and types. By simulating the natural complexes, the scaffold protein can locate the active sites of various enzymes and play a key role in the metabolic process.

This review introduces the research progress in studying the two main strategies of compartmentalization and substrate channel for organisms to manipulate multi-enzyme reactions. The enzyme immobilization strategies mediated by scaffolds and the discovered enzyme cascade reactions related to scaffold proteins will also be discussed. In the design of multi-enzyme complexes, the role and influence of linkers on catalytic efficiency are still debatable. The effect of various linkers on the fusion protein (fusion enzyme) and its possible mechanism was elaborated. Furthermore, the design and selection of the linker were discussed. So far, various protein assemblies have been developed as multi-enzyme scaffold platforms. This review is expected to provide a more theoretical basis for modifying multi-enzyme complexes and broaden their application in synthetic biology. The scaffold protein promises new insight into the assembly and consequences of the multi-enzyme complexes.

## Metabolic channeling in multi-enzyme complexes

**Fig. 1:**
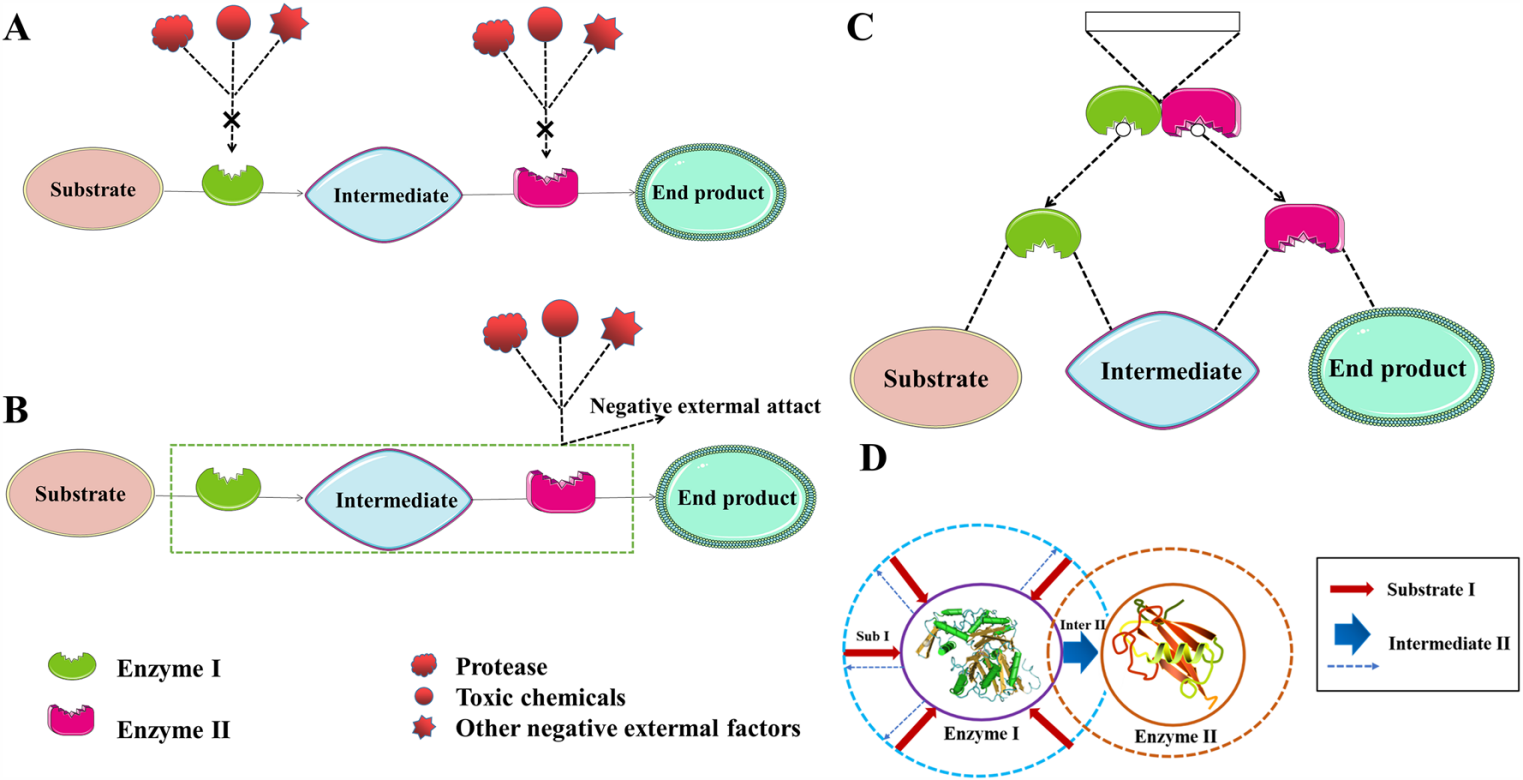
Schematic diagram of multi-enzyme catalysis. **A** Ideal multi-enzyme catalytic system would inhibit negative external attack and promote the transfer of intermediates between adjacent enzymes. The design and construction of multienzyme catalytic systems often confront challenges, the intermediate product produced by the first step reaction, enzyme I may diffuse back to the overall environment, inhibit the intermediate enrichment around enzyme II and reduce the activity. The multi-enzyme catalytic systems may be degraded by protease, produce toxic substances, or encounter other external attacks. **B** Scheme of a compartmentalized cascade reaction, Compartmentalization for in vivo multi-enzyme reactions could inhibit the negative external attack toward multi-enzymes by encasing them in a robust and semi-permeable membrane. **C** Substrate channeling for in vivo multi-enzyme reactions could facilitate the transfer of intermediates between neighboring enzymes by shortening the inter-enzyme distance. **D** Simulation diagram of substrate channel effects in the catalytic process of cascading enzymes in metabolic compartments

Enzyme cascades occur naturally in most metabolic pathways within cells, ensuring the integrity of enzyme-catalyzed transformations that mimic chemical processes (Ricca et al. 2011). The enzymes involved in these metabolic pathways play a role in an organized way, carry out multi-step reactions, and maintain the growth and survival of cells. According to widespread opinion, most cascades in metabolic pathways are spatially bound by non-covalent protein–protein interactions (PA 1987; You 2012). Cascade enzymes are close to each other in space in the metabolic pathway of organisms and form multi-enzyme complexes through non-covalent interaction to form metabolic compartments (Tsitkov 2019). Sun et al. utilized cascade enzyme reactions to improve biocatalytic efficiency and increase product yield (Sun et al. 2022). As we know, many multi-enzyme reactions must be well-regulated in organisms to achieve effective metabolic processes. The ideal multi-enzyme catalytic system should be capable of preventing external attacks and promoting the transfer of intermediates between adjacent enzymes (Fig. 1A) (Shi 2018). Compartmentalization and substrate channel are two main strategies for organisms to manipulate multi-enzyme reactions.

### Strategies for in vivo multi-enzyme reactions through compartmentalization

Compartmentalization involves isolating multi-enzymes or enzyme systems with tandem reaction features in a compartment through a semi-permeable membrane (Fig. 1B). The semi-permeable membrane evolved for compartmentalization to inhibit protease and toxic chemical external attack, ensuring that multi-enzyme reactions could proceed stably and controllably (Avalos et al. 2013). Despite its intuitive utility in containing toxic reactants and enhancing the concentration of unstable intermediates, quantitative models were just recently published. Stanislav et al. studied a basic model for a compartmentalized two-enzyme cascade reaction in a well-mixed, steady-state regime. Cascade reactions, according to the model can be characterized by the number of molecules of each enzyme rather than concentrations. Furthermore, compartment capacity has only a minimal impact on homeostasis. We were able to define and solve the compartment optimization problem using the model in circumstances when the intermediate was volatile or poisonous (Tsitkov, 2019). Compartmentalization aims to create a suitable micro-environment against adverse external factors and improve enzyme stability.

### Strategies for in vivo multi-enzyme reactions through substrate channeling

Studies have shown that the substrate channel effect and synergistic mechanism are the main reasons for the significantly improve of the catalytic efficiency of multi-enzyme complexes after the self-assembly of multiple catalytic elements in some natural metabolic pathways (Sweetlove and Fernie 2018; Sheng et al. 2022). The substrate channel effect is a process in which the product of an enzyme is directly transferred to the adjacent cascade enzyme. It is not restricted by equilibrium (Fig. 1C), and the catalytic efficiency of the cascade enzyme is significantly high (Spivey HO 1999). Wu et al. identified eight enzymes by in vivo cross-linking and mass spectrometry, demonstrating the existence of Krebs circulating metabolites. Two models of the wild-type mitochondrial malate dehydrogenase–citrate synthase–aconitase (mMDH–CS–ACON) complex and a disymmetric octamer consisting of two mMDH dimers, one CS dimer, and two ACON monomers are proposed using the distance constraints derived from crosslinking. Analysis of the surface electrostatic potential of the model shows that the rearrangement of the surface charge pattern in protein–protein binding results in the formation of a continuous positively charged region at its interface. Thus, the electrostatic channels formed by enzyme association are favorable for direct transport of intermediates between active sites (Wu and Minteer 2015). As a result, directed transport of negatively charged substrates from one active site to the next would be possible with minimal diffusion to the body phase of the cell. Notably, the enzyme complex can be considered integral, so the overall apparent activity of the enzyme complex is linearly related to its concentration. The acceleration factor of enzyme complex to enzyme mixture increased with the total enzyme concentration. As shown in the simulation diagram of the metabolic compartment in Fig. 1D, the concentration of local enzyme and substrate increased. Furthermore, the substrate channel effect in the process of cascade enzyme catalysis is used to direct the reaction intermediate from the first enzyme active site to the second enzyme active site without maintaining the balance in the solution, resulting in the realization of a multi-enzyme catalytic system and improved catalytic efficiency and stability of the enzyme (Shi 2018; Khobragade et al. 2021).

In the cascade enzyme reaction, the lag time of transient product formation of the fusion protein is shorter than that of the free enzyme, indicating a channel transfer of metabolites in the fusion protein (Henrik Pettersson 2001). Kang et.al emphasizes the modular assembly of metabolic enzymes, manipulating the physical location of enzymes without changing the activity or foundation of the enzymes holds promise to produce high yield strains (Kang et al. 2019). Constructing fusion protein between cascade enzymes is the simplest way to facilitate substrate channeling, Laura et al. used a fusion of S. *carnosus* monomeric fructose-1,6-bisphosphate aldolase and *C. freundii* CECT 4626 homodimeric dihydroxyacetone kinase with five amino acid linkers. The overall reaction rate was much higher in the reaction catalyzed by the fusion enzyme than in the reaction catalyzed by the native non-fused enzymes (Iturrate 2009). However, substrate channeling in fusion proteins may also be absent. An exploration of the kinetics of the coupled reaction catalyzed by a fusion protein of L-galactosidase and galactose dehydrogenase found insufficient kinetic evidence to support the proposal that fusion proteins catalyze the formation of galactolactones from lactose via mechanisms involving galactose channels (Henrik Pettersson 2001). The area of metabolic channeling has been well-reviewed (Zhang 2011; You 2012; Wheeldon, 2016). While exploring the literature, it was evident that substrate channeling existed in dynamic metabolons of enzymes, such as TIM, ALD, and FBP (Beeckmans S, 1993). When the substrate level was low, the channelization of the substrate was more significant (You, 2012). Some articles have highlighted the latest progress of multi-enzyme reactions, mainly focusing on the substrate channel strategy based on DNA and protein scaffold (Schoffelen and Hest 2013; Wheeldon 2016; Buddingh' BC 2017; Nakata et al. 2019; Tsitkov 2019; Wang et al. 2023b). The method by which synthetic multi-enzyme complexes increase product synthesis has long been debated. Idan et al. studied the concept of "metabolic channels" generated by the rapid transfer of intermediate substrates through free diffusion between two enzymes on nanoscale scaffolds through simulation and mathematical models. The weak attractive interaction between substrate molecules and scaffolds creates a "virtual compartment" and greatly accelerates initial production (Idan and Hess 2013). Hess et al. highlight that the enhancements are not caused by the proximity of the enzymes, but rather by the scaffold altering the enzyme characteristics (Zhang and Hess 2017). However, Meng et al.'s study suggests that the catalytic efficiency of enzyme complexes is higher than that of enzyme mixtures, which may be caused by changes in enzyme properties in protein scaffolds, or by the construction of enzyme complexes that increase reaction rates by reducing activation energy. It cannot be ruled out that the reason for the increased activity of enzyme complexes is the proximity effect of enzymes (Meng et al. 2019). As a result, the process of accelerated product synthesis in spatial tissue protein scaffolds is still being debated, and additional study is needed to prove it. Aside from increasing reaction rates through substrate channels in the complexes, many potential benefits of these complexes include the protection of unstable substrates, the avoidance of adverse equilibria and kinetics, the prevention of substrate competition between different pathways, the regulation of metabolic fluxes, the mitigation of toxic metabolite inhibition, and so on (Zhang 2011; Wheeldon 2016).

## Strategies of enzyme immobilization mediated by a scaffold

So far, there are several various effective methods to bring enzymes close to each other. Nevertheless, there is limited control over how components are built. Affinity tags, DNA scaffolds, and protein scaffolds have been used for selective immobilization of proteins (Muller and Niemeyer 2008; Hernandez and Fernandez-Lafuente 2011; Schoffelen and van Hest 2012).

### Selectively fixed to the solid bracket using an affinity tag

The use of affinity tags to immobilize functional proteins on solid carriers is an attractive method in protein microarray technology (Freitas et al. 2022). For, example, materials such as glass and acrylic glass functionalized with nickel (II) ions have also been employed in affinity-mediated oriented immobilization of His-tagged recombinant proteins (Kulsharova et al. 2018; Takahashi et al. 2018). Among the commonly used fusion protein tags, glutathione S-transferase (GST) proteins have been indispensable tools for protein–protein interaction studies and have extensive applications in recombinant protein purification and reversible protein immobilization. Zhou et al. reported that *Schistosoma japonicum* GST (*sj*GST) fusion protein was selectively immobilized by irreversibly and covalently modified by a small pyrimidine probe with sulfonyl fluoride reaction group (Zhou et al. 2014) The result strongly indicated that glutathione S-transferase (GST) protein is indispensable among the commonly used fusion protein tags in studying protein interaction. It has been widely used in the purification of recombinant proteins and the immobilization of reversible proteins (Viswanathan et al. 2013; Voelker and Viswanathan 2013). Covalently binding tags are most suitable for long-term protein immobilization, but can only bind naturally to protein-based materials.

### Enzyme immobilization strategies mediated by DNA scaffold

The ability of DNA to self-assemble nanostructures, along with the accuracy of nanoparticle localization on DNA scaffolds, presents a potential strategy for the self-organization of composite nanostructures (Winfree et al. 1998; Park et al. 2008b; Li et al. 2018; Nakata et al. 2019; Komarala et al. 2022). Muller et al. reported for the first time on the production of heterodimerase complexes of Glucose Oxidase (GOX) or Horseradish peroxidase (HRP) through DNA-directed assembly of enzyme oligonucleotide conjugates. Although the spatial requirements of the bulky enzyme hindered the quantitative formation of GOX–HRP–DNA ternary complexes, the total enzyme activity of the complexes significantly increased, in which GOX and HRP were mobilized in direct proximity on a complete DNA carrier (Muller and Niemeyer 2008). Orthogonal module adaptors are a promising approach for constructing explicit protein assemblies in vitro and possibly in the cell, with spatial and orientational control of enzymes on the DNA scaffold. Nguyen et al. studied a series of modular linkers on sequence specific DNA binding domains and self-linking protein labels to achieve quick kinetics and high loading rate of cross-linking reactions, with high orthogonality with a single target address (Nguyen et al. 2017). Nonetheless, the amplification cost of DNA scaffolds may be too high compared with protein scaffolds.

### Enzyme immobilization strategies mediated by the protein scaffold

While most affinity tags or binding modules fused with enzymes are used to fix solid scaffolds, some components have been built on protein scaffolds. Many anaerobic cellulose-degrading bacteria degrade plant cell walls, producing extracellular complexes of large molecules called cellulose bodies (Bayer, 2004; Schoffelen and van Hest 2012). Compared with other co-localization techniques, protein scaffolds have some advantages. First, the minimum distance between the active site on the protein scaffold allows for effective substrate channels, because metabolic intermediates are more likely to undergo sequential reaction steps rather than diffusion (Deng et al. 2020). When expressed within cells, the protein scaffold maintains a high local concentration, while the total cell level remains relatively low. Due to the faster conversion of unstable compounds, toxic intermediates have a lower chance of damaging cells (Ivarsson 2021). In addition, the protein scaffolds can be applied in vitro and in vivo. The most direct method is to express and purify different interacting proteins separately. Then, they can bind in vitro for cascade reactions. Another option is to express all interacting proteins in the same host cell and react in vivo. A major advantage of protein scaffolds is that they can easily target the entire complex to specific locations. This is usually achieved by adding a localization tag to the scaffold protein (Wang and Yu 2012; Chen et al. 2022). Furthermore, protein scaffolds allow for control and optimization of spatial organization, stoichiometry, and enzyme proximity, which is particularly important when flux balance correction is required (Whitaker and Dueber 2011; Cai et al. 2022). The main problem of protein scaffolds is the time-consuming protein labeling and the associated genetic engineering. Furthermore, the creation of these synthetic compounds is more time-consuming and sophisticated than the other procedures discussed (Vanderstraeten and Briers 2020). Table 1 provides an overview of reported examples of multi-enzyme complexes based on the protein scaffolds.

**Table 1:** Summarized the use of multi-enzyme complexes based on SpyTag/SpyCatcher, cohesin–dockerin and SH3–PDZ–GBD for enzyme cascade

Scaffold protein	Location	Function	References
SpyTag/SpyCatcher	In vivo	Achieved higher sugar conversion yield	(Jia et al. 2017)
	In vivo	Increased the oil synthesis	(Zhong et al. 2022a)
	In vivo	Improved the catalytic efficiency	(Wang et al. 2023a, b)
Cohesin–dockerin specificity	In vitro	Improved catalytic efficiency	(You 2012)
	In vitro	Increased the reaction rate of bioelectricity generation	(Meng et al. 2019)
	*E. coli* cell surfaces	Enhanced the power density and operational stability	(Cai et al. 2022)
SH3, PDZ, GBD domain and ligand	In vivo	Increased the production of mevalonate	(Dueber et al. 2009)
	In vivo	Increased the production of GBGA	(Pham et al. 2016)

**Fig. 2:**
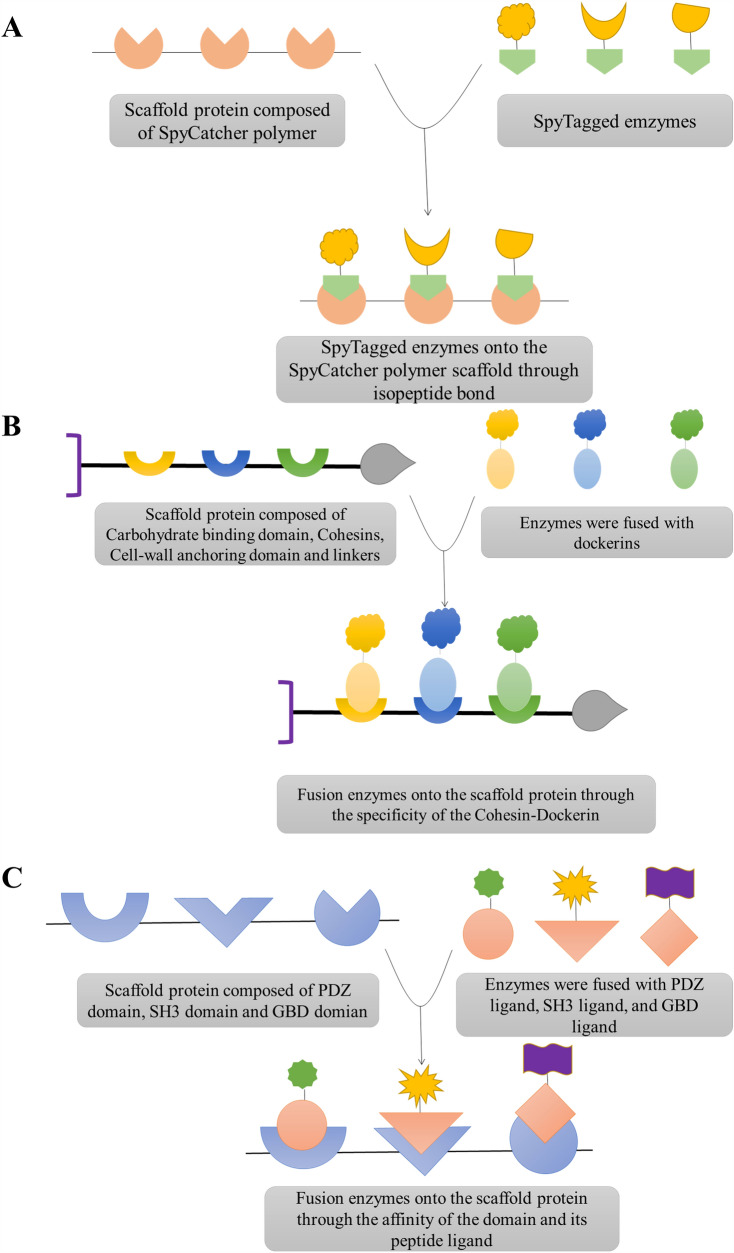
Protein scaffold systems mainly used for assembling multi-enzyme complexes. **A** SpyTag/SpyCatcher system. **B** Cohesin–dockerin specificity. **C** SH3, PDZ, GBD domain and ligand

Scaffold proteins naturally participate in signal cascades. They provide docking sites for various protein members of signal cascades, thus smoothing corresponding interactions and functions (Rapali et al. 2017; Srour et al. 2022). Furthermore, the multi-enzyme complexes mediated by scaffold protein decrease the loss of intermediates, decrease overall transit time, and reduce product feedback inhibition due to the proximity of catalytic sites. Scaffold proteins consist of several protein modules separated by linkers to produce skeleton molecules or these specific modules fuse with proteins to self-assemble to form the scaffold skeleton. The high-affinity interaction between the scaffold modules and the modules fused with the enzyme allows for the controlled assembly of specific enzymes. The enzyme is assembled into the scaffold by fusing it to a domain orthogonal to the scaffold module. Three main types of interaction domains are used to assemble complexes: the SpyTag–SpyCatcher system, the cohesin–dockerin specificity, and SH3, PDZ, GBD domain, and ligand (Fig. 2).

**Fig. 3:**
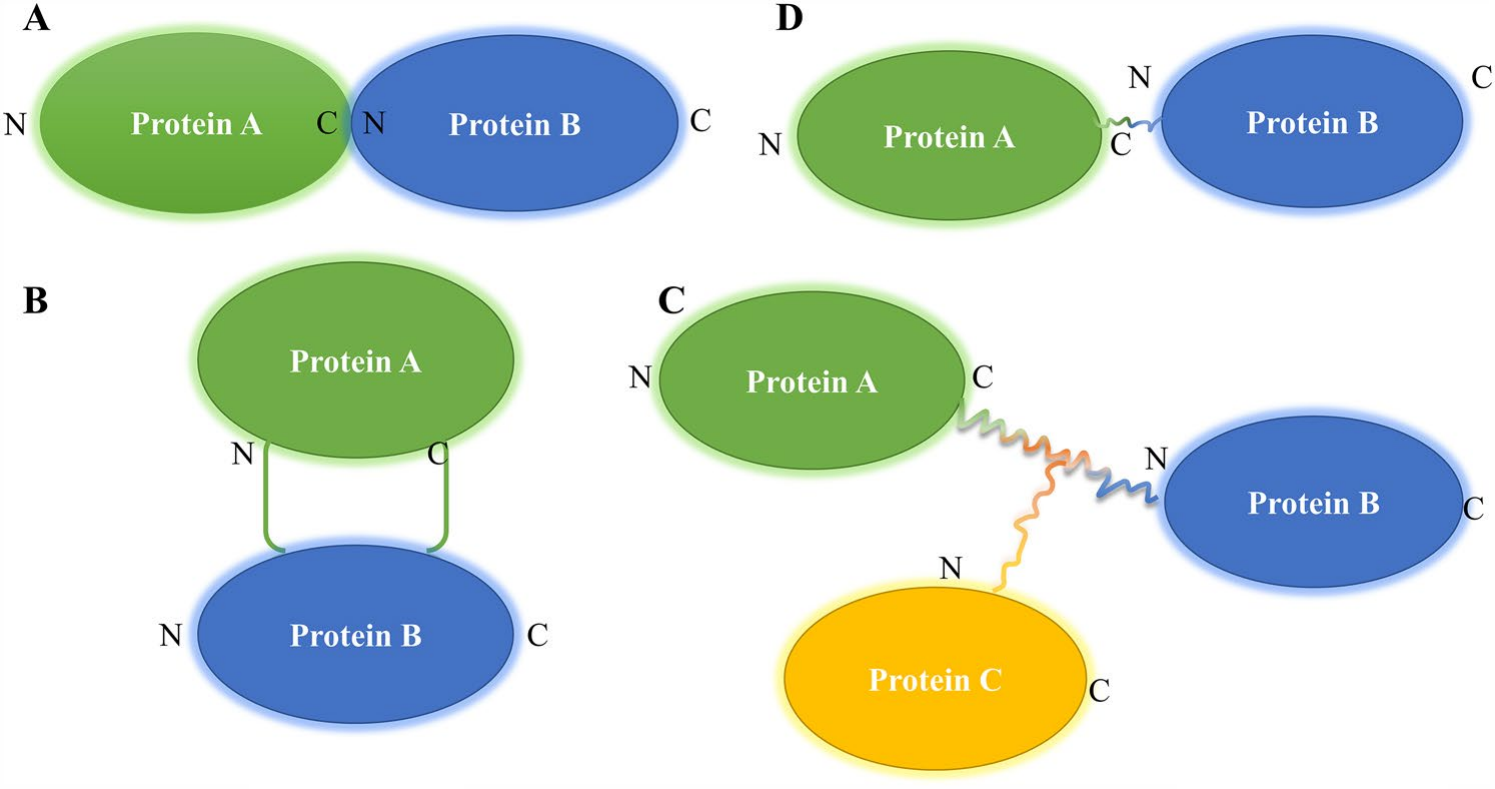
Several strategies for constructing fusion protein. **A** End-to-end fusion; **B** insertional fusion; **C** branched fusion; **D** end-to-end fusion with a linker. (In the figure, N and C represent the N-terminal and C-terminal of the protein, respectively)

The fusion protein comprises at least two structural domains encoded by individual genes that are already connected, thus being transcribed and translated as a unit to produce a peptide (Wang et al. 2018). Theoretically, the structure of scaffold proteins and fusion proteins is very similar and can be obtained through gene fusion. The difference is that sites on the scaffold proteins can bind to each other, forming a multi-enzyme complex. As a product of recombinant DNA technology, fusion protein plays a vital role in biochemistry, biotechnology, and biomedical. Fusion protein products can obtain different functions from different components by fusing two or more protein domains(Chen et al. 2013b; Arai 2021) There are mainly four ways to construct fusion proteins: end-to-end fusion, insertional fusion, branched fusion, and linker fusion (Fig. 3) (Huston JS 1988; Nobuhide Doi 1999; Guntas and Ostermeier 2004; Hirakawa and Nagamune 2009). End-to-end fusion is the simplest and most widely used for fusion protein. However, misfolding or inappropriate protein interactions may destabilize protein structures and affect activity levels (Hong et al. 2006). Our previous investigation, we found that inapposite assembly owing to the spatial interference of the element domain may result in the loss of activity in the end-to-end fusion protein (Karp and Oker-Blom 1999; Hong et al. 2006; Yang et al. 2015). The construction of insertion fusion protein has a significant potential value in catalytic regulation and biosensors (Ataka and Pieribone 2002; Ribeiro et al. 2019). However, some critical issues remain, such as how to select two proteins and how to choose the insertion site, so that they can have regulatory effects after fusion (Huang 2012). Branching fusion makes the domain more balanced, thus reducing interference and steric hindrance. However, its use remains limited due to the need for additional in vitro modification steps. The most effective way to overcome these problems is to introduce a linker between different domains, which can act as an appropriate bridge between them while keeping them separate but connected (Huang et al. 2021) As an integral part of the recombinant fusion proteins, we found that a suitable linker can provide a stable connection of fusion proteins without disturbing their original biological activity or interactions, proving highly valuable in their construction, expression, stability, solubility, and biological activity (Fan et al. 2015; Arai 2021).

#### SpyTag/SpyCatcher system

**Fig. 4:**
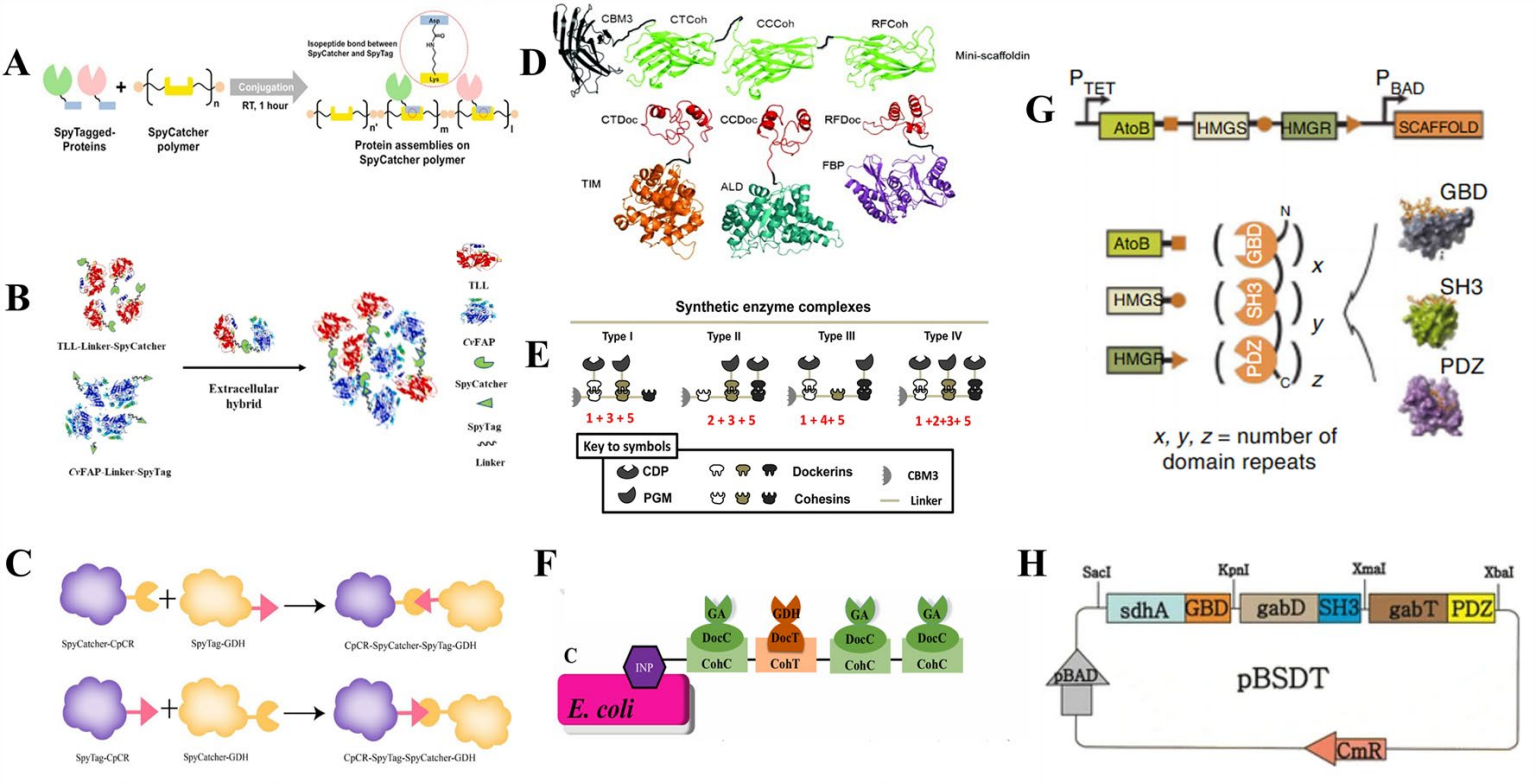
Protein scaffold for enzyme assembly. **A** Preparation of biosensing module capable of detecting the model antigen ovalbumin with SpyTagged Nanoluciferase and SpyTagged protein G using SpyCatcher polymer (Jia et al. 2017). **B** Preparation of the oil synthesis with TLL–CvFAP (Zhong et al., 2022a). **C** Regenerating NADPH with a special structure and excellent catalytic activity with CpCR–SpyCatcher–SpyTag–GDH (Wang et al. 2023a, b). **D** Assembly of the TIM, ALD, and FBP fused dockerin in the mini-scaffoldin (You, 2012). **E** Generating bioelectricity from cellodextrin with CDP–PGM enzyme complex (Meng et al. 2019). **F** Preparation a starch-oxidizing bioanode based on displaying a sequential enzyme system of GA–GDH on *E. coli* cell surfaces in a precise way using cohesin–dockerin interactions (Cai et al. 2022). **G** Synthetic scaffolds built from modular protein–protein interaction domains (Dueber et al. 2009). **H** Increased the production efficiency of GABA with the protein–protein interaction domain of GBD, SH3, and PDZ scaffold architecture (Pham et al. 2016)

The SpyTag/SpyCatcher system has become a vital technology for multi-enzyme systems. Proteins are designed using SpyTag/SpyCatcher technology, in which protein components can be expressed individually and used as building blocks to generate complex protein assemblies (Kajiwara et al. 2021). The SpyTag/SpyCatcher system derives from the CnaB2 domain, where Asp117 on SpyTag and Lys31 on SpyCatcher can spontaneously form covalent bonds combining protein assembly and chemical reactions that are genetically encoded chemical reactions. The SpyTag/SpyCatcher system is versatile and offers more possibilities for protein bioconjugation (Sutherland et al. 2019; Yanase et al. 2023). A polymer of SpyCatcher was recently applied to fabricate protein assemblies using SpyTagged enzymes (Fig. 4A). Jia et al. used the SpyTag/SpyCatcher system to generate a biosensing module capable of detecting the model antigen ovalbumin. The SpyCatcher polymer was used as a scaffold to assemble the SpyTagged Nanoluciferase and SpyTagged protein G, which was then used as a biological probe for ELISA, allowing ovalbumin detection (Jia et al. 2017). The system's usefulness in assembling biomolecules to construct biological probe modules is due to protein clustering reactions amplifying luminescence signals. Zhong et al. demonstrated a self-assembly strategy based on SpyTag/SpyCatcher to overcome diffusion limitations (Zhong et al. 2022b). In this study, TLL–Linker–SpyCatcher based on the lipase from *Thermomyces lanuginosus* and CvFAP–Linker–SpyTag based on the fatty acid photodecarboxylase from *Chlorella variabilis* was designed as the multi-enzyme complex (TLL–CvFAP). Compared with double-free enzyme catalysis, the efficiency of TLL–CvFAP in oil synthesis was increased by about 50%, and the storage stability of TLL–CvFAP after self-assembly was also significantly improved (Fig. 4B) Another research group has demonstrated that the multi-enzyme complex based on the SpyTag/SpyCatcher system improves catalytic efficiency (Fig. 4C) (Wang et al. 2023a, b). The stereoselective carbonyl reductase (CpCR) and glucose dehydrogenase (GDH) successfully fused with SpyCatcher and SpyTag to form two double enzyme self-assembled clusters, named CpCR–SpyCatcher–SpyTag–GDH and GDH–SpyCatcher–SpyTag–CpCR. CpCR–SpyCatcher–SpyTag–GDH showed better activity and efficiently converted ethyl 2-oxo-4-phenylbutyrate (OPBE) to ethyl(R)2-hydroxy-4-phenylbutanoate ((R)-HPBE), while regenerating NADPH with a unique structure and excellent catalytic activity, improving the catalytic efficiency of the enzyme (Wang et al. 2023a, b). Overall, these examples demonstrate the practicality of the SpyTag/SpyCatcher system in biotechnology. Since it was proposed, the SpyTag/SpyCatcher system has been widely used in synthetic biology, nanobiotechnology, protein engineering, and other fields. However, it is more widely used in developing two enzymes or nanoparticle vaccines, which is difficult to assemble multiple enzymes (Zhong et al. 2022a).

#### Cohesin–dockerin specificity

The current protein scaffolds are mainly used in soluble enzyme systems. Cellulosome is a distinctive example of a naturally occurring protein scaffold system comprising structural backbone (scaffoldins), where cellulases have been localized via dockerin–cohesin interactions. Dockerins and cohesins also seem to function differently and may be overexpressed in surrogate host bacteria. They can be fused or cross-linked to various enzymes or the components of affinity systems, such as binding proteins, nucleic acids, and other biologically active materials (Takagi M, 1993; Tokatlidis K, 1993). Due to its highly ordered structure and better catalytic efficiency, cellulosomes have potential applications in various biorefineries (Hu and Zhu 2019). In 1994, Bayer et al. demonstrated that dockerins or cohesins could be fused or conjugated to protein A, antibodies, lectins, DNA, etc., to form hybrid biomolecules. Moreover, it has become clear that the interaction is characterized by a different set of principles (Edward A. Bayer 1994) It has been analyzed and verified that the cohesin–dockerin interaction is nature's most efficient protein–protein interaction (Fierobe 2001; Mechaly 2001). Constructing a multi-enzyme complex based on the interaction between cohesins and dockerins is stronger and easier than that based on the nucleic acid (Quin et al. 2017).

So far, many researchers have used the scaffold protein and interactive domains from natural cellulosomes to co-localize multiple enzymes. The scaffold protein carrying cohesins and the fusion enzyme carrying dockerins were constructed using DNA recombination technology. After expression and purification, the desired multi-enzyme complex was assembled in vitro*.* According to studies by Chun You and coworkers, they construct the dockerin-containing enzymes Triosephosphate isomerase (TIM), aldolase (ALD), and fructose 1,6-bisphosphatase (FBP) by adding one dockerin from the *C. thermocellum* CelS, *C. cellulovorans* EngE, and *R. flavefaciens* ScaA at their C termini. They discovered that the activities of dockerin-containing enzymes TIM–*Ct*Doc, ALD–*Cc*Doc, or FBP–*Rf*Doc in the presence of mini-scaffoldin were similar to those of dockerin-free enzymes TIM, ALD, and FBP (You 2012). The addition of dockerins to the C terminus of TIM, ALD, and FBP did not affect their activities. This means that the complexes of these three enzymes may face each other to form dimers with TIM, ALD, and FBP (Fig. 4D). Many studies have tested the apparent kinetic parameters of enzyme complex and free enzyme mixture, showing that the catalytic efficiency of enzyme complex is higher than that of enzyme mixture.

The effects of enzyme orientation in enzyme complexes are yet to be experimentally described. They represent an important future research area for studying the molecular mechanisms of enzyme complexes. The multi-enzyme complexes constructed based on scaffold proteins are mostly the specific of cohesin–dockerin and linker. For instance, based on the high-affinity and high specific interaction between cohesins and dockerins from natural cellulosomes, Meng et al. constructed four self-assembled synthetic enzyme complexes containing cellodextrin phosphorylase (CDP) and phosphoglucomutase (PGM) with different spatial organizations for generating bioelectricity from cellodextrin (Meng et al. 2019). The results showed that the in vitro biological system containing the optimal CDP–PGM enzyme complex exhibited significantly higher current density (3.35 times) and power density (2.14 times) than the corresponding biological system containing a free CDP and PGM mixture (Fig. 4E). To explore a high-performance starch/O2 enzymatic biofuel cell, Cai et al. prepared a starch-oxidizing bioanode based on displaying a sequential enzyme system of glucoamylase (GA) and glucose dehydrogenase (GDH) on *E. coli* cell surfaces using cohesin–dockerin interactions (Cai et al. 2022). They discovered that the co-displayed GA&GDH-*E. coli* bioanode-based enzymatic biofuel cells outperformed the randomly mixed system regarding power density and operational stability, exhibiting a maximum open-circuit voltage of approximately 0.74 V and the largest P +  + max of 30.1 ± 2.8 μW cm^−2^ as well as good operational stability with GA&GDH (3:1)–*E. coli.* (Fig. 4F) The Cohesin–dockerin showed high-affinity interactions (Kd = 10^−9^–10^−12^ M) and were species-specific, ensuring the availability of an abundance of orthogonal interaction pairs present in the natural reservoir (Haimovitz et al. 2010). However, research has shown that since promiscuity binding is possible, the specificity of cohesin–dockerin should always be verified. The main drawback of using the specificity of cohesin–dockerin to assemble enzymes is that their interaction is calcium-dependent, and the calcium concentration can affect intracellular assembly (Edward A. Bayer 1994; Vera 2021). The best concentration of Ca^2+^ assembled in our laboratory is 2–10 mM. The assembly mechanism of natural cellulosomes has not yet been clarified, which may hinder the expression of protein scaffolds composed of cellulosome building blocks. Nevertheless, mucin-based scaffolds are an excellent technology for in vitro assembly and bacterial cell surface display of protein scaffolds that assemble enzyme cascades.

#### SH3, PDZ, GBD domain, and ligand

The interaction domains used in protein scaffolds mostly come from naturally occurring multi-enzyme complexes, namely, cellulosomes. Theoretically, any natural domain with an interacting partner can be incorporated into a synthetic protein scaffold. The PSD-95 MAGUK family scaffold protein is a multi-domain organizer of synaptic transmission and contains three PDZ domains followed by the SH3-GK domain tandem. This domain structure allows for the coordinated assembly of protein complexes consisting of neurotransmitter receptors, synaptic adhesion molecules, and downstream signaling effectors (Rademacher et al. 2019). The family of postsynaptic density protein-95/disks large/zonula occludens-1 (PDZ) protein domains is one of the most common protein–protein interaction modules in mammalian cells. The general function of the PDZ domain is to aggregate proteins in the appropriate cell compartment, thereby facilitating scaffolding, signaling, and transport events (Chi et al. 2012; Kossmann et al. 2021). A growing number of SH3 domain–ligand interactions are being described, all of which involve the conserved peptide-binding surface of SH3 but are structurally distinct from canonical docking of SH3 ligands containing consensus motifs. The SH3 domain is a small protein interaction module composed of five strands connected by three loops and a short 3_10_ helix. Early pioneering research revealed how the ligand sequence PxxP is regulated by two different xP dipeptide binding pockets on the surface of SH3 (Ren et al. 1993; Saksela and Permi 2012).

A typical example is the study of mevalonate production using a synthetic scaffold (GBD, SH3, and PDZ scaffold) strategy by Dueber et al. in 2009 (Dueber et al. 2009). In this study, the protein–protein interaction ligands, such as GBD, PDZ, and SH3, were fused with acetoacetyl-CoA thiolase (AtoB), hydroxy-methylglutaryl-CoA synthase (HMGS), and hydroxy-methylglutaryl-CoA reductase (HMGR), respectively (Fig. 4G). The authors were able to increase the yield of mevalonate by 77-folds by optimizing the molecular ratio of GBD, PDZ, and SH3 (1:2:2). The synthetic protein scaffold was used to produce gluconate (Moon et al. 2010), resveratrol (Wang and Yu 2012) and gamma-aminobutyric acid (Pham et al. 2016), with a significant increase in product yield. Pham et al. directed metabolic flux to a new gamma-aminobutyric acid (GABA) production pathway by introducing a synthetic scaffold (GBD, SH3, and PDZ scaffold) strategy in *E. coli.* In this study, the protein–protein interaction ligands, such as GBD, SH3, and PDZ, were fused with succinate dehydrogenase (SdhA), succinate–semialdehyde dehydrogenase (GabD), and GABA aminotransferase (GabT), respectively (Fig. 4H). The enzyme–ligand complexes were then co-expressed with the protein–protein interaction domain of GBD, SH3, and PDZ scaffold architecture. With the introduction of the synthetic scaffold, 0.75 g/L of GABA was produced from 10 g/L of glucose at 30 °C and pH 6.5. Finally, the concentration of GABA increased by 15.4%, indicating that the inactivation of competitive metabolic pathways in *E. coli* can enhance the GABA concentration in mutant strains. This new pathway can be further improved to increase the production efficiency of GABA and applied to the industrial GABA process (Pham et al. 2016). Nature has provided us with a much larger array of interacting domains. The major advantage of SH3, PDZ, GK domain, and ligand is that their interactions are not calcium-dependent and may improve in vivo assembly. However, the interaction between these modules is not as strong as the high affinity cohesin–dockerin interactions. The K_d_ value of domain ligand interaction is 10^–7^ M for the SH3 domain and its ligands and 10^–6^ M for the PDZ and GBD domains and their respective ligands (Whitaker and Dueber 2011; Vanderstraeten and Briers 2020).

## Design and selection of linker between the fusion protein

Both scaffold proteins and fusion enzymes require a linker to connect. Scientific research has shown that linker is an inherently disordered protein lacking the tertiary structure, which not only exists as a connecting region between functional modules but also has essential biological functions (Espinoza-Fonseca et al. 2010; Meng et al. 2015; Rozycki 2016). Linker refers to a polypeptide between two fused enzymes or domains. Its length ranges from several to hundreds of amino acid residues. It is generally believed that it is not directly related to the catalytic process of the enzyme (Gokhale and Khosla 2000; George and Jaap 2002; Wriggers, 2005) Furthermore, the role and influence of the linker on catalytic efficiency in the design of multi-enzyme complexes is still debatable (Smith 2017). The presence of a linker between the two domains of natural enzymes suggests that such "fusion" proteins are produced as part of a natural evolutionary process (Argos 1990; Gokhale and Khosla 2000). The two domains of cellulase are connected by a long and highly glycosylated linker, primarily rich in proline and hydroxyproline. Bacterial cellulose linker is rich in Pro and Thr and is entirely composed of the repetitive sequence of Pro–Thr. Bacteria have about 100 amino acid residues, but fungal cellulase linker is rich in Pro, Ser, and Thr with only 30–40 amino acid residues, their number is less than that of bacteria (Cavaco–Paulo et al., 1999). Table 2 provides an overview of the characteristics of different types of linkers.

**Table 2:** Characteristics of different types of linkers

Linker		Fusion protein	Function	References
Type	Sequence			
Flexible linker	GGGGS	HSA–IFN-α2b	Improve biological activity	(Zhao et al. 2008)
		TD1–hEGF	Inhibit the permeation activity	(Ruan et al. 2014)
	(GGGGS)_2_	DFR–LAR	Improve catalytic efficiency	(Sun et al. 2021)
	(GGGGS)_3_	IL-2–PfCelTOS	Enhance structural stability	(Shamriz et al. 2016)
		Tax–gp21–gp46–gag	Increase expression level	(Kabiri et al. 2020)
	(GGGGS)_4_	CotB–Tm1350	Enhance enzyme activity	(Ullah et al. 2017)
		CotB–DSM	Enhance enzyme activity and stability	(Chen et al. 2017)
Rigid linker	EAAAK	CBDs–CP05	Enhance structural stability	(Ma et al. 2022)
		Fluc–LRE	Improve stability and continuity	(Sun et al. 2018)
	(EAAAK)_2_	DFR–LAR	Improve catalytic efficiency	(Sun et al. 2021)
	(EAAAK)_3_	Glu–Xyl	Increase catalytic efficiency	(Lu and Feng 2008)
	(EAAAK)n (n = 2,3)	VP1–SpyTag	Enhance conjugation efficiency	(Boonyakida et al. 2023)
	EAAAK (PAPAP)n (n = 1,2)	HSA–CocH1	Prolong biological half-life	(Cai et al. 2019)

Whether the two components in the fusion protein can form the correct spatial structure and give better play to their biological activities depends on the linker connecting the two components in the fusion protein (Meng et al. 2015; Ma et al. 2022). The recombinant fusion protein requires the linker inserted into the fusion protein not to interfere with the target protein’s functions (Huang 2012; Chen et al. 2013a; Jiang 2021). Robinson and Sauer found that the linker sequence's composition may significantly impact the folding stability of fusion protein (Robinson and Sauer 1998). To avoid secondary structural elements, we emphasize that the design and selection of a linker sequence often require careful consideration. Unfortunately, there are no trustworthy selection criteria in linker design. Most linker design and selection processes still rely on intuition to a great extent. This intuitive process of designing and selecting a linker often leaves much uncertainty, especially in the case of long linker sequence selection. However, significant advances have been made in protein secondary structure prediction based on the primary sequence (Barton 1995; Jones 1997). There is no denying that the understanding of sequence structure correlation is still limited. Thus, the design and selection of linker sequences are crucial for the construction, expression, stability, and functional activity of fusion proteins. Fusion protein construction often requires linkers for prolonged conformation, extended stability, and enzyme activity. Many related investigations and studies have been conducted on the design and selection of linker sequences (Crasto C J 2000).

### Design of linker for the fusion proteins

With the wide-ranging study on the linker of natural multi-domain proteins and recombinant fusion proteins, the researchers conceived the intention of setting up a database and proposed linker design tools to rationally design Linkers based on the desired properties of the fusion proteins. Such a program is called LINKER. Meanwhile, with the intention of the rational design of linkers for domain fusion, the web-based project http://www.ibi.vu.nl/programs/linkerdbwww/ performed by the Centre for Integrative Bioinformatics VU at the Vrije University of Amsterdam also offers a database containing various confirmed linkers (Kabsch and Sander 2006; Chen et al. 2013a). Using bioinformatics technology, deriving molecular structure from homologous modeling, and improving the genetic algorithm to design Linker is a relatively scientific design method (James et al. 2005; Arunachalam et al. 2006; Dietmann et al. 2006). These tools and databases help us implement high-throughput linkers for the required attributes from extended candidates. Nonetheless, search algorithms have limitations in predicting the most appropriate linker in practice (Fan et al. 2015). Although many researchers are absorbed in the design of linkers, they have not yet found an efficacious method. As a result, further optimizing the selection of linkers based on experimental analysis is unavoidable.

### Selection of linker for the fusion proteins

**Fig. 5:**
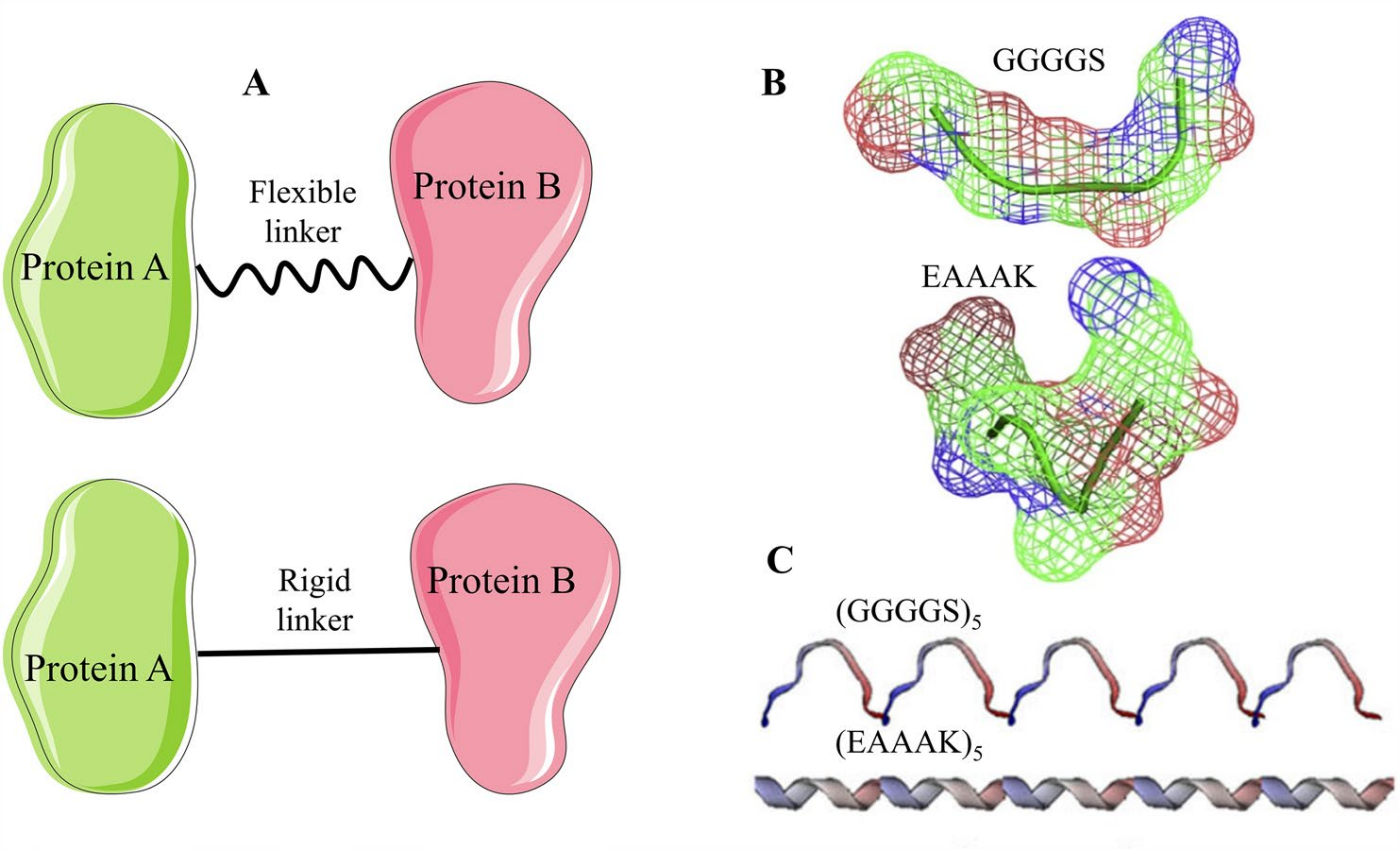
Schematic diagram of the linker. **A** Fusion proteins are connected by the linker. **B** 3D structure of the flexible linker (GGGGS) and rigid linker (EAAAK), which were obtained from SynLinker (Meng et al. 2020). **C** Molecular dynamics simulations of a selected group of linkers (Li et al. 2016)

Factors such as flexibility, composition, conformation, length, and hydrophilicity of the amino acid should be considered when selecting a linker for the fusion proteins (Robinson and Sauer 1998; Arai R, 2001; Chen et al. 2013b; Fan et al. 2015; Yang et al. 2015; Li et al. 2016; Huang et al. 2021). According to the flexibility of the linker, they can be divided into flexible linkers and rigid linkers (Fig. 5). Whether the linker is flexible or rigid will affect the direction of the fusion protein. Many studies have shown that the flexibility of a linker is closely related to the function of the fusion protein. Therefore, selecting an appropriate linker is particularly important in the fusion protein (Maeda et al. 1996; Wriggers, 2005).

#### The flexible linker

The flexible linker is a soft and flexible linker with the non-polar amino acid glycine (Gly) or a polar amino acid, such as serine (Ser) or threonine (Thr) as the main component (Argos 1990). Gly ensures the freedom of peptide skeleton conformation to the greatest extent. Thus, the protein has sufficient spatial folding to obtain the original biological activity, but this connection will free the functional proteins at both ends from rigid constraints. Nevertheless, Ser and Thr can form hydrogen bonds with water molecules, so it can ensure the stability of the linker in an aqueous solution, reducing the adverse reactions between protein regions and the linker. The most commonly used flexible linker is primarily composed of Gly and Ser residues, with the (GGGGS)n (generally n ≤ 6) sequence proposed by Huston et al. being the most common example (Huston JS, 1988). By adjusting the number of repeats n and optimizing the length of the GS linker, the functional domain can be properly separated, or the function between domains can be maintained, which has almost become a "universal linker" (Hu et al. 2004; Trinh et al. 2004; Yun and Shen 2006; Hong et al. 2008). Remarkably, more than 11 amino acids are required to ensure the biological activity of the fusion protein. Although the flexible linker has no rigid structure, it can serve as a passive linker to maintain the distance between functional domains.

A fusion of DFR and LAR was successfully employed, significantly improving the flux toward flavan-3-ols. Chen et al. used the esterase–DSM gene from C. *thermocellum* and the cotB gene from B. *subtilis* to generate CotB–DSM fusion proteins with different lengths and types of the linker to investigate the optimal linker conformation. They discovered that fusion proteins with linker (GGGGS)_4_ were the most thermostable at 80 °C. Meanwhile, the fusion proteins with longer flexible linkers showed increased activity, while those with longer rigid linkers showed decreased activity (Chen et al. 2017). A glycine-rich linker is more flexible than a linker of the same length composed of non-glycine residues. Jawad et al. reported that after inserting a (GGGGS)_4_ linker between CotB and Tm1350 from *Thermotoga maritime* MSB8, the catalytic efficiency of the fusion protein was 1.29 times higher than the activity of the original under optimum temperature and pH (Ullah et al. 2017). Ruan et al. showed that the permeation activity of TD1–hEGF, a fusion protein composed of TD1 and human epidermal growth factor (hEGF) connected with the flexible linker (GGGGS), can be inhibited by the energy inhibitor, rotenone or oligomycin (Ruan et al. 2014).

#### The rigid linker

The rigid linker comprises amino acid residues that easily form a stable secondary structure and are not easy to bend. The most commonly used rigid linker in recombinant fusion proteins is (EAAAK)n (n ≤ 6) or proline-rich sequences (XP)n with X designating any amino acid, preferably Ala, Lys, or Glu (Evans et al. 1986; Yun and Shen 2006; Nurmamet et al. 2009; Huang et al. 2021). The presence of Pro can increase rigidity and effectively separate protein domains (Turner et al. 1993; Wriggers 2005; Arai 2021). Studies have shown that proline-rich connexin (XP) n is often used as a linker, because it has been proven to resist protease degradation and is used in many natural multi-domain proteins (Mojgan et al. 2007). As suggested by Ma et al., the presence of EAAAK can make the exosomal capture peptides (CP05) and collagen-binding domains (CBD) of the fusion protein have a fixed distance in space, so that they do not interfere with each other, and the fusion protein has higher structural stability (Ma et al. 2022). Sun et al. inserted a rigid linker EAAAK between the Firefly luciferase (Fluc) and luciferin-regenerating enzyme (LRE) to improve luminescence production. The dual enzymes showed higher stability and continuity in signal generation (Sun et al. 2018). Lu et al. used a rigid linker (EAAAK)_3_ to fuse beta-glucanase (Glu) and xylanase (Xyl), which increased the catalytic yield by 31% and 26.2%, respectively (Lu and Feng 2008). Boonyakida et al. inserted a linker (EAAAK)n (n = 2,3) between the SpyTag and the VP1 protein to increase surface exposure of the SpyTag on the NoV-LPs. They discovered that the conjugation efficiency of the VP1–SpyTag with the linker (EAAAK)n (n = 2,3) improved from ∼15–35 to ∼50–63% based on the densitometric analysis (Boonyakida et al. 2023). Cai et al. redesigned albumin-fusion cocaine hydrolase CocH1 (TV-1380) to extend its biological half-life. The half-life of the fusion protein (HSA–CocH1) without a linker was 14 ± 2 h, while the fusion protein HSA–CocH1 with a rigid linker extended the biological half-life, with HSA–EAAAK–CocH1 and HSA–PAPAP–CocH1 having a half-life of 17 ± 2 h and HSA–(PAPAP)_2_–CocH1 having a half-life of 18 ± 3 h (Cai et al. 2019).

The (GGGGS)_2_ and (EAAAK)_2_ linkers between dihydro flavonol 4-reductase (DFR) and leucoanthocyanidin reductase (LAR) were proved by Sun et al. to be the best choice for afzelechin (AFZ) and catechin (CAT) production (Sun et al. 2021). Fusion protein brings enzyme-active sites nearby for consecutive reactions. Kabiri et al. evaluated the effects of the helix and flexible linkers on the expression levels of multi-epitope chimeras containing four epitopes of Human T Lymphotropic Virus Type 1 (HTLV-1). Their data showed that inserting flexible (GGGGS)_3_ linkers between chimera epitopes significantly increased expression levels, while chimeras containing helix (EAAAK)_5_ linkers had lower expression levels (Kabiri et al. 2020). The absence of a β-carbon in glycine might allow the polypeptide backbone to access dihedral angles that are energetically forbidden for other amino acids, and the ability of serine to form hydrogen bonds might allow the formation of new stabilizing interactions in the native state.

Compared with (GGGGS)n, (EAAAK)n has the advantage of forming a relatively stable secondary structure and providing a relatively stable and controllable isolation effect for two connected domains. However, when the number of repeat units is increased, the distance between domains changes little, and the isolation effect varies significantly across systems (Arai et al. 2004). The 3D structures of the flexible linker (GGGGS) and rigid linker (EAAAK) were obtained from SynLinker (Fig. 5). Because (EAAAK)n is not a stretched conformation, the possibility of protease attack is somewhat reduced, making the fusion protein more stable. The extended conformation of (GGGGS)n may make it the cleavage site of protease, resulting in the instability of fusion protein. Previous research has shown that the α-helical content of rigid linker (EAAAK)n increased proportionally with the number of repeats, whereas more flexible linker (GGGGS)n resulted in more random conformations of the fusion protein (Arai R, 2001). In a recent investigation by Li et al., attention was drawn to the number of repeats n in (EAAAK)n. With the increased number of repeats, the rigid linker was exposed to more helical conformations and hydrogen bonds in the simulated conformation (Li et al. 2016). The hydrogen bond in the helical conformation helps improve the linker's rigidity (Karle et al. 1997; Igor et al. 1999).

The length of the amino acid sequence in the linker must be designed to keep the active site closer together and strengthen the substrate channel effect (Iturrate 2009). The length of the linker directly affects the spacing of functional proteins. Too long or too short a linker will affect the stability and activity of fusion protein. If the linker is too long, it will increase the sensitivity of the linker to protease, resulting in the instability of the fusion protein. While too short linker will often make the parent domains of the fusion protein too close, thus affecting their respective functions (Malin et al. 2001). The length of the linker varies significantly, generally ranging from 6 to 59 amino acid residues (Black et al. 1994; Pham et al. 2010; Song HY 2014). The linker with 10 to 22 amino acid residues is generally believed to be more effective but also reported for 4 to 44 amino acids or 6 to 27 amino acids (Malin et al. 2001; Gall et al. 2004). Experiments by George et al. showed that solvent accessibility improved with the increase in the length of the linker, indicating that longer linkers were more likely to be exposed to the solvent. Furthermore, with the increase in the length of the linker, the average hydrophobic linker decreased, suggesting that the longer linkers were more hydrophilic than shorter linkers, so they were more likely to be exposed to aqueous solvents (George and Jaap 2002).

Robinson et al. examined and compared the linker of different lengths, and found that the most stable protein has a linker with 19 residues. Increasing or decreasing a handful of amino acids would reduce the stability of the protein (Robinson and Sauer 1998). Although long linkers may contribute to immune reaction, those with less than 15 amino acids have little effect on protein folding and function. According to the study by G.G. Yang and coworkers, the length of linkers only affected the activity of rHSA-N-ONC (N = 0, 5, 10, and 15) but not its expression (Yang et al. 2015). The short linkers have been successfully applied in many fusion proteins. They also have many defects, such as being vulnerable to protease attack, which in turn leads to the degradation of proteins. Moreover, the short linkers fail to avoid the spatial interference of the regions (Maeda et al. 1997; Robinson and Sauer 1998; Arai et al. 2004). Kavoosi et al. have proved that the expression level, proteolytic stability, and thermodynamic stability of the fusion protein expressed in recombinant *E. coli* can be decided by the chemical properties and the length of the linker (Kavoosi 2007). Bouin et al. also showed that enzyme orientation and linker length are crucial for the performance of fusion enzymes (Bouin et al. 2023). The design and selection of valuable linkers should not be based solely on proteolytic stability. The thermodynamic stability and spatial localization of the fusion protein may also change the expression level, thus affecting the overall performance of the fusion label.

Both scaffold proteins and fusion enzymes require a linker to connect. The linker must be flexible enough to allow the natural folding of each protein. The selection of an appropriate linker is vital for the distance and orientation between fusion proteins and scaffold proteins.

## Conclusion

From the examples discussed in this review, it is clear that the spatial organization of the enzyme cascade reaction on the appropriate protein scaffold has attracted attention because of its vital role in the substrate channel to obtain the best catalytic efficiency and provide a stable micro-environment. The substrate channeling effect, the number and order of the cohesins in a scaffold protein, the number and orientation of enzymes on the kinetic performance of synthetic enzyme complexes, and the design and selection of linkers between cohesins and fusion enzymes are crucial factors of further investigations in the structural orientation among cascade enzymes and metabolite transfer among them. Many more experiments are still required to get a complete picture of the interactions between these factors.

## Data Availability

The authors confirm that the data supporting the findings of this study are available within the review.
